# Report on Current Experience of ASAPS Membership and Management of Cosmetic Tourism Complications

**DOI:** 10.1093/asjof/ojz009

**Published:** 2019-04-09

**Authors:** Ali A Qureshi, Daniel J Gould, W Grant Stevens, James Fernau

**Affiliations:** 1 private plastic surgical practice in Marina del Rey, CA; 2 Department of Plastic and Reconstructive Surgery, Keck Hospital of USC, Los Angeles, CA; 3 Surgery and Director of the Aesthetic Surgery Fellowship, University of Southern California School of Medicine, Division of Plastic Surgery, Los Angeles, CA; 4 Private practice in Pittsburgh, PA

## Abstract

**Background:**

Cosmetic tourism is an expanding industry with increasing scrutiny in the public domain of complications and patient safety issues. The health and financial implications for patients are large and deserve further investigation.

**Objectives:**

The aim of this study was to understand the experience of the American Society for Aesthetic Plastic Surgery (ASAPS) members treating medical tourism patients with complications who returned to the United States for secondary management.

**Methods:**

A 20-question survey was administered electronically in August 2018 to ASAPS members with voluntary participation. Questions about surgeon experience, the nature of complications, type of initial surgery, and subsequent management were asked. Responses were tabulated and percentages of response choices were calculated and reported.

**Results:**

Ninety-three responses were received from the 1611 physician ASAPS members (5.8% response rate). More than half of respondents had seen 2 to 5 patients in the last 12 months with a complication from cosmetic tourism. The most common procedure that patients had done abroad was abdominoplasty. The most common complication was infection caused by Gram-positive organisms, managed on an outpatient basis without surgical intervention. Involvement of an ASAPS member led to successful resolution of complications in the vast majority of patients. Estimated costs out of pocket for management of complications were most commonly between $1001 and 5000.

**Conclusions:**

While the experience of ASAPS members is as varied as the complications faced by cosmetic tourism patients, the vast majority of complications is infectious and can be managed on an outpatient basis successfully with the involvement of an ASAPS member. Further collaborative efforts both domestically and internationally can help improve patient safety for cosmetic tourism patients.

Medical tourism for cosmetic and aesthetic surgery (“cosmetic tourism”) is an expanding industry with increasing scrutiny in the public domain of complications and patient safety issues.

Multiple authors have demonstrated the rise of medical tourism, with estimates of 3 to 15 million Americans seeking care outside the United States for their medical treatment.^1-3^ The International Society of Aesthetic Plastic Surgery (ISAPS) surveyed over 1300 members and found 56% agreed that cosmetic tourism is growing and 25.4% perceived this as a dangerous trend.^4^ In 2010, Evans reported more than 50% of surgeons at the Transatlantic Innovations Meeting in Paris, France, thought medical tourism was affecting their practice and 75% believed related complications should not be covered by national healthcare.^5^ This could represent a loss of over $375 billion to the American medical system^6^ according to a report by the Deloitte group. More importantly, medical tourism represents a risk to patients in several other areas. Firstly, it represents a potential risk in terms of immediate outcomes, spreading resistant organisms,^7^ increased threats to informed consent,^8^ and postoperative follow-up^9^ in addition to a milieu of legal and regulatory concerns.^10,11^ The American Society for Aesthetic Plastic Surgery (ASAPS) and ISAPS have created guidelines to help patients avoid poor outcomes in cosmetic tourism.

Numerous studies have demonstrated the high incidence of poor aesthetic outcomes and high rates of complications following surgery outside the United States^1,9^ and much has been written to increase the quality of aesthetic surgery and to set standards to decrease risk.^12,13^ One study demonstrated an extremely high rate of wound infections and wound healing complications with 86% of the patients relying on Massachusetts Medicaid for health insurance coverage to assist in their care.^14^ The cost of poor outcomes associated with medical tourism is often carried by the medical systems in the patient’s home state. In one article, the cost associated with medical tourism for the NHS was estimated between 6000 and 10,000 pounds per patient.^15^ No doubt health insurers and public health plans are bearing the brunt of the burden for poor outcomes related to medical tourism.^16-19^

Why do patients pursue cosmetic tourism if there are known increased risks? Without question, cost is a key factor, with several studies describing the average cost of a breast augmentation in the United States at $6000 vs $2200 in India for instance.^2,20^ Patients may be attracted to destination surgery due to the difference in cost when that difference is much less than the cost of the travel to the destination. Patients may be lured by differences in technology or surgical procedural skill as in the case until recently in transgender surgery in Thailand.^2,21-23^ There may be a role for ethical engagement in medical tourism under the right context and circumstances, but better informational tools and dissemination of this information must be done for patients.^24^

Despite the increasing number of plastic surgery patients seeking procedures abroad, there is a paucity of data concerning outcomes, follow-up, and complication rates. We report the results of a survey of ASAPS members about their experience treating medical tourism patients with complications who returned to the United States for secondary management.

## METHODS

A 20-question survey (SurveyMonkey, San Mateo, CA) was developed by the Patient Safety Committee of the American Society for Aesthetic Plastic Surgery to assess the current experience of surgeons with management of cosmetic tourism complications (Appendix A, available as Supplementary Material online at www.asjopenforum.com). Cosmetic tourism was defined as cosmetic surgery done outside of the United States. Questions about surgeon experience, the nature of complications, type of initial surgery, and subsequent management were asked. The survey was sent in August 2018 to 1611 physicians, all active members of the American Society for Aesthetic Plastic Surgery (ASAPS) and collected over 21 days with two separate e-mail reminders sent to members. Responses were anonymous and collected into an Excel (Microsoft, Redmond, WA) spreadsheet. Because responses were anonymous, it was not possible to know whether an individual surgeon completed the survey more than once, although ASAPS members were only asked to complete the survey once. Questions either had single responses or options for multiple responses depending on the nature of the question. Responses were tabulated and percentages of response choices were calculated and reported.

## RESULTS

Ninety-three responses were received from the 1611 physician ASAPS members (5.8% response rate). There were 997 total opens of the survey and 131 clicks to complete the survey. Respondents of the survey were from the West (31.5%), Midwest (15.2%). Northeast (27.2%), and South (26.1%) with the majority in solo private practice (68%). 92.5% of respondents had seen or evaluated a patient in their practice who traveled outside of the country for an aesthetic surgery procedure.

**Figure 1.**
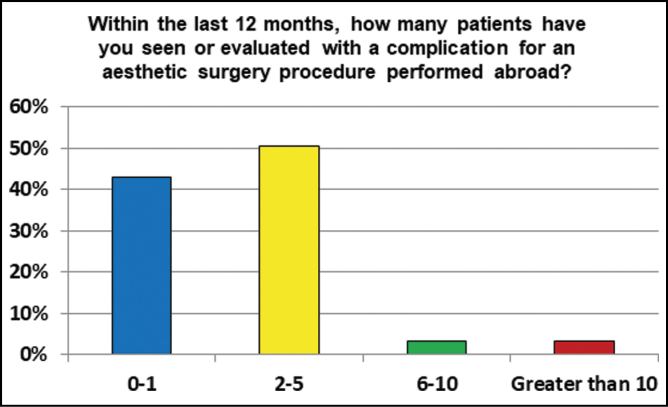
The number of patients seen in the last year in each practice with complications from international surgery.

**Figure 2.**
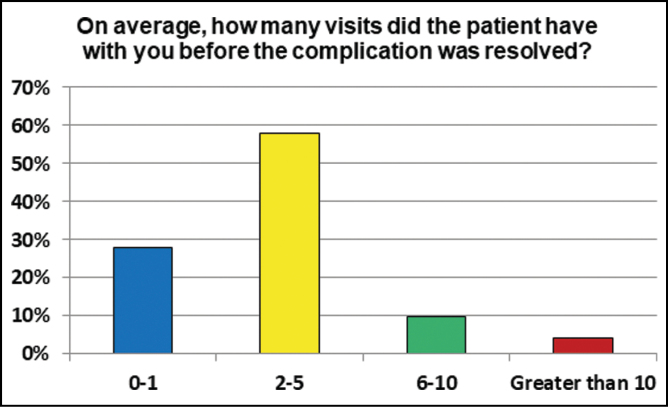
The number of visits required to resolution of the complications suffered by international patients.

Within the last 12 months, 50.5% respondents had seen or evaluated 2 to 5 patients with a complication for an aesthetic surgery procedure performed abroad (Figure 1). 43% had seen 0 to 1 patients, 3.2% saw 6 to 10 patients and 3.2% saw more than 10 patients with complications from cosmetic tourism. 58.1% reported that on average 2 to 5 visits were needed before the complication was resolved, while a minority (4.3%) needed more than 10 visits (Figure 2).

**Figure 3.**
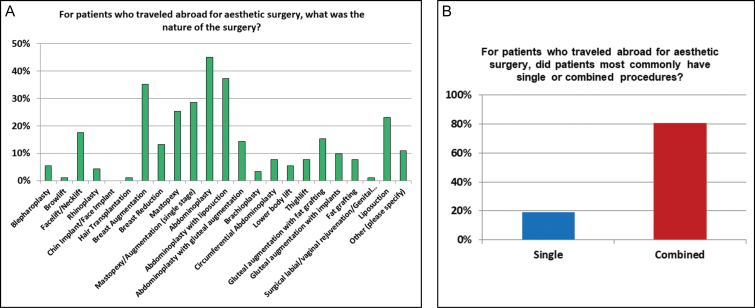
The types of procedures seen from the international patients (A) and the preponderance of multiple surgical procedures in these international cosmetic tourist surgical centers (B).

The five most common surgeries performed abroad included abdominoplasty (*n* = 41), abdominoplasty with liposuction (*n* = 34), breast augmentation (*n* = 32), single-stage mastopexy augmentation (*n* = 26), and mastopexy (*n* = 23) (Figure 3A). The least common procedures were browlift (*n* = 1), hair transplantation (*n* = 1), and genital rejuvenation (*n* = 1). For patients who traveled abroad for their survey, 81% had combined procedures while 19% had single procedures (Figure 3B).

**Figure 4.**
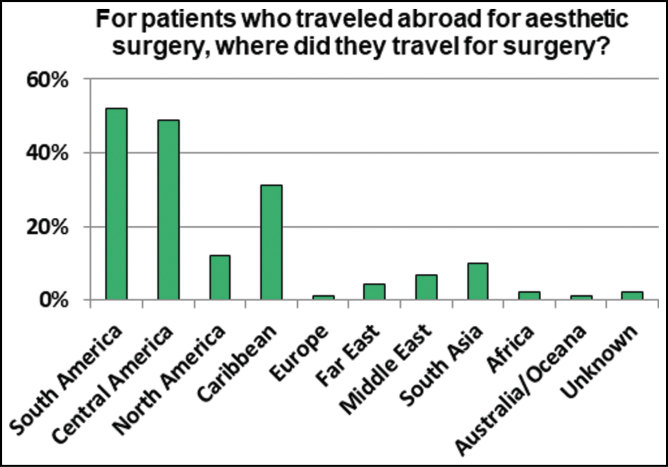
The many locations patients had traveled for their cosmetic procedures.

The most popular destinations for cosmetic tourism were South America (*n* = 47), Central America (*n* = 44), Caribbean (*n* = 28), North America (*n* = 11) and South Asia (*n* = 9) (Figure 4). Only 1 patient that was evaluated was reported to have travelers medical insurance.

**Figure 5.**
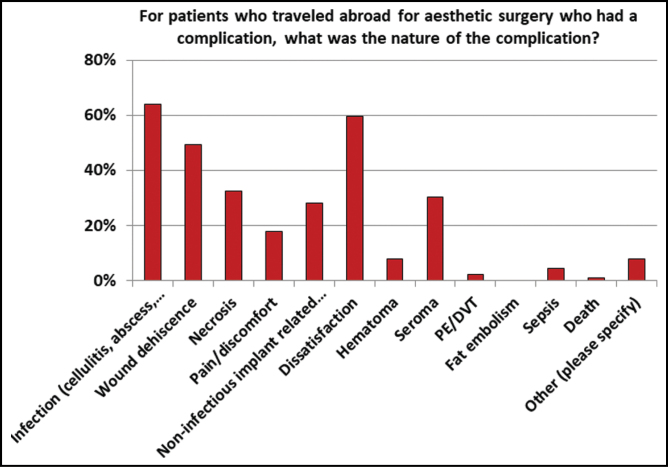
The diversity of the nature of the different complications seen in these cosmetic tourists.

The most common complications were infection (cellulitis, abscess, necrotizing infection) (*n* = 57), dissatisfaction (*n* = 53), wound dehiscence (*n* = 44), necrosis (*n* = 29), and seroma (*n* = 27). Only one respondent reported a patient death. In general, infectious complications were more commonly treated with non-operative management (58.1%) vs operative (41.9%). Wound dehiscence was more commonly treated with nonoperative management (72%) vs operative (28%). Necrosis, however, was more commonly treated with operative management (69.7%) than nonoperative (30.3%). Seroma was treated more commonly with nonoperative management (60%) than operative (40%). Dissatisfaction was more commonly treated with operative management (62.5%) than nonoperative (37.5%) (Figure 5).

For patients who traveled abroad and had a complication, they most commonly presented 14 to 30 days (32.6%) after surgery to respondents of the survey. 26.7% presented between 7 and 14 days and 26.7% presented after 30 days from surgery.

When infectious complications were further examined, the majority (60%) were treated as outpatient with oral antibiotics and without surgery. 20% required inpatient admission and IV antibiotics along with surgery, although 12.3% required inpatient admission and IV antibiotics alone. Management with surgery alone was only 6.2%. Additionally, management with an interventional radiology procedure was only reported in one instance (1.5%).

The bacteria causing infectious complications were unknown in 46% of cases. Gram-positive (33.3%), Gram-negative (9.5%) and atypical bacteria (6.4%) made up the rest. No fungal infections were reported.

**Figure 6.**
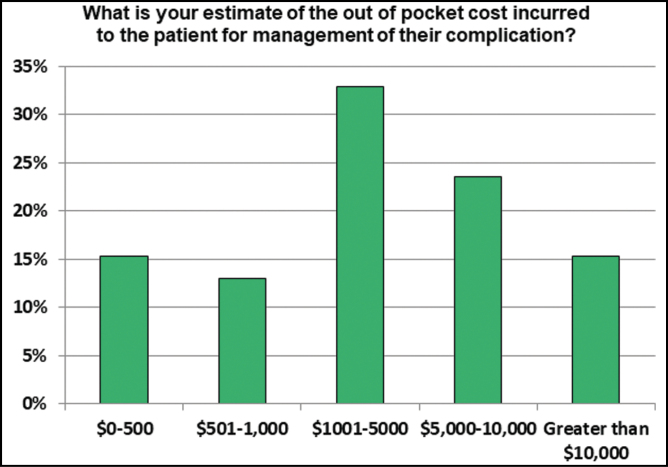
The perceived or estimated cost to the patient of these complications.

For those patients who had a complication, the problem was resolved successfully after the involvement of the respondent in 91.6% of the time. Estimated costs out of pocket for management of complications was most commonly (32.9%) between $1001 and 5000. 15.3% of respondents reported costs greater than $10,000 (Figure 6).

50.6% of respondents reported that the patients who had cosmetic procedures performed returned for a secondary procedure unrelated to the complication. 97.7% of respondents said that complications they managed did not lead to a legal issue requiring their involvement. Only 2.3% said their management led to a legal issue requiring their involvement.

86.1% of respondents said they would be willing to submit information to an ASAPS-sponsored database to track complications and their management. When asked if reluctant to treating patients who have had cosmetic surgery abroad, the majority (43.8%) said they were not reluctant. 23.6% had concerns about medical liability about assuming a patient with a complication and 23.6% expressed a philosophical opposition that the patient is seeking care only after having a complication. 7.9% expressed reluctance based on financial concerns to the patient.

## DISCUSSION

Cosmetic tourism is increasing in popularity and remains a concern of the Patient Safety Committee of the American Society for Aesthetic Plastic Surgery. In an effort to understand the current experience of ASAPS members with patients who have traveled abroad for aesthetic surgery, a survey was created and sent to members. Although only a small percentage of members responded, this is the only existing snapshot of member experiences with cosmetic tourism patients to date.

Several salient features were observed from the survey. Taken together, the majority of respondents are private practice members who saw at more than one patient with a complication after cosmetic tourism that was resolved with 2 to 5 visits. The nature of the complication was most commonly infectious, which is similar to findings previously reported,^1^ with complications presenting most often in the 14- to 30-day time period. The majority of infectious complications were managed with outpatient antibiotics and did not warrant surgical intervention. When bacteria was identified, it was most commonly Gram-positive organisms. However, when patients presented with a complication of necrosis, these patients more commonly required operative intervention. The involvement of an ASAPS member surgeon led to the resolution of the complication in the overwhelming majority of patients. When patients were dissatisfied from their cosmetic surgery abroad, it often required operative intervention and many patients returned to the surgeon for another procedure unrelated to their initial surgery elsewhere.

In addition the patient safety issues and health risk borne by the patient, the financial implications of complications in these patients cannot be underestimated and have been explored in detail elsewhere.^14^ We found that most commonly management of the complication cost less than $5000 to the patient. However, a sizeable number of patients did spend more than $10,000 for managing their complication.

There are limitations to the present study including the low response rate. However, it is possible that only a small number of ASAPS members have taken care of cosmetic tourism patients with complications and thus the response rate is low. Alternative methods of survey administration may have yielded different response rates. Additionally, the present study can only present responses from questions asked and not unasked questions. The survey only was given to plastic surgeons and not patients, and it would be valuable to understand the patient’s perspective better in another study. Indeed, understanding the full extent and experience of cosmetic tourism complications will require further work and collaboration with other professional organizations and societies. Guidelines by ASAPS and ISAPS are available online for patients.^25,26^

The present survey study is a starting point to understanding the problems faced by cosmetic tourism patients and the experience of ASAPS members caring for them. The majority of respondents who are managing these complications are plastic surgeons in private practice who belong to the community of ASAPS and therefore may benefit from ASAPS guidelines and recommendations that may come from studies such as the present one. This study lays the foundation from which the Patient Safety Committee can work to improve the safety of cosmetic tourism patients seeking the help of ASAPS members. Improved consensus guidelines and networks of plastic surgeons locally and abroad can potentially help, but dissemination of information to lay will also be important. Additionally, databases for ASAPS members to submit information as cosmetic tourism patients are treated and managed can help prospective data collection and analysis. This could help identify clusters of infectious complications and guide management strategies since most members seeing these complications are in private practice and not in large, academic centers where centralization of information and outbreaks may be more easily identified.

## CONCLUSIONS

While the experience of ASAPS members is as varied as the complications faced by cosmetic tourism patients, the vast majority of complications are infectious and can be managed on an outpatient basis successfully with the involvement of an ASAPS member. There are significant costs to patients that experience complications and may or may not require surgery, even when patients are dissatisfied with their results from procedure performed abroad. Further collaborative efforts both domestically and internationally can help improve patient safety for cosmetic tourism patients.

## Disclosures

The authors declared no potential conflicts of interest with respect to the research, authorship, and publication of this article.

## Funding

The authors received no financial support for the research, authorship, and publication of this article.

## Supplementary Material

Supplementary Appendix A
