# Supplementary material for: Report on Current Experience of ASAPS Membership and Management of Cosmetic Tourism Complications
Source: Aesthet Surg J Open Forum. 2019 Apr 9;1(2):ojz009. doi: 10.1093/asjof/ojz009 (PMC7671244; doi:10.1093/asjof/ojz009)
Supplement: Supplementary Appendix A [file ojz009_suppl_supplementary_appendix_a.docx]

**Appendix A.** Medical Tourism Survey for ASAPS Membership

| **Question** | **Answers** |
| --- | --- |
| 1. Where in the United States do you practice? | - West - Midwest - Northeast - South |
| 1. What is the nature of your practice? | - - Solo private practice   - Group practice   - Academic   - Hospital system employee |
| 1. Have you seen or evaluated a patient in your practice who traveled outside of the country for an aesthetic surgery procedure? | - - Yes   - No |
| 1. Within the last 12 months, how many patients have you seen or evaluated with a complication for an aesthetic surgery procedure performed abroad? | - 0-1 - 2-5 - 6-10 - Greater than 10 |
| 1. On average, how many visits did the patient have with you before the complication was resolved? | - 0-1 - 2-5 - 6-10 - Greater than 10 |
| 1. For patients who traveled abroad for aesthetic surgery, what was the nature of the surgery? | - Blepharoplasty - Browlift - Facelift/Necklift - Rhinoplasty - Chin Implant/Face Implant - Hair Transplantation - Breast Augmentation - Breast Reduction - Mastopexy - Mastopexy/Augmentation (single stage) - Abdominoplasty - Abdominoplasty with liposuction - Andominoplasty with gluteal augmentation - Brachioplasty - Circumferential Abdominoplasty - Lower Bodylift - Thighlift - Gluteal augmentation with fat grafting - Gluteal augmentation with implants - Fat grafting - Surgical labial/vaginal rejuvenation/Genital rejuvenation - Liposuction - Other:______ |
| 1. For patients who traveled abroad for aesthetic surgery, did patients MOST COMMONLY have single or combined procedures? | - Single - Combined |
| 1. For patients who traveled abroad for aesthetic surgery, where did they travel for surgery? | - South America - Central America - North America - Caribbean - Europe - Far East - Middle East - South Asia - Africa - Australia/Oceania - Unknown |
| 1. Did any of the patients you evaluated for a complication have traveler’s medical insurance? | - Yes - No |
| 1. For patients who traveled abroad for aesthetic surgery who had a COMPLICATION, what was the nature of the complication? | - Infection (cellulitis, abscess, necrotizing infection) - Wound dehiscence - Necrosis - Pain/discomfort - Non-infectious implant related issues (exposure, capsular contracture, malposition) - Dissatisfaction - Hematoma - Seroma - PE/DVT - Fat embolism - Sepsis - Death - Other |
| 1. Was the management of the complication, operative or non-operative/conservative management? | - Operative - Non-operative/Conservative   *Answers will be for each complication in 9 in table format with boxes to check operative or non-operative for each complication* |
| 1. For patients who traveled abroad for aesthetic surgery who had a COMPLICATION, how soon after surgery did they present to you with a complication? | - 1-2 days - 3-5 days - 5-7 days - 7-14 days - 14-30 days - > 30 days |
| 1. If you treated a patient with an infection, how was this managed? | - - Outpatient with oral antibiotics, no surgery   - Outpatient with IV antibiotics, no surgery   - Outpatient with oral antibiotics and surgery   - Outpatient with IV antibiotics and surgery   - Outpatient with oral antibiotics and interventional radiology procedure   - Outpatient with IV antibiotics and interventional radiology procedure   - Inpatient with IV antibiotics, no surgery   - Inpatient with IV antibiotics and surgery   - Surgery alone   - Other |
| 1. If you answered YES to above, which bacteria was identified? | - - Unknown   - Gram positive   - Gram negative   - Atypical mycobacterial   - Fungal   - Other:____ |
| 1. For those patients who had a complication, was the problem resolved successfully after your involvement? | - - Yes   - No |
| 1. What is your estimate of the out of pocket cost incurred to the patient for management of their complication? | - $0-500 - $501-1,000 - $1,001-5,000 - $5,001-10,000 - Greater than $10,000 |
| 1. Have any of the patients who had cosmetic procedures performed abroad returned to you for a secondary procedure unrelated to the complication? | - Yes - No |
| 1. Have any of the complications you managed led to a legal issue that required your involvement? | - Yes - No |
| 1. In the future, would you be willing to submit information into an ASAPS sponsored database to track complications and their management for patients who traveled abroad for aesthetic surgery procedures? | - Yes - No |
| 1. If you are reluctant to treating patients who have had cosmetic surgery abroad, what is your primary reluctance: | - Not reluctant - Financial concerns to the patient - Medical liability assuming a patient with a complication from abroad - Philosophical opposition that patient is seeking your care only after having a complication - Other: |
